# Modulation of Global Gene Expression by Aneuploidy and CNV of Dosage Sensitive Regulatory Genes

**DOI:** 10.3390/genes12101606

**Published:** 2021-10-12

**Authors:** Shuai Zhang, Ruixue Wang, Cheng Huang, Ludan Zhang, Lin Sun

**Affiliations:** 1 Beijing Key Laboratory of Gene Resource and Molecular Development, College of Life Sciences, Beijing Normal University, Beijing 100875, China; 202031200005@mail.bnu.edu.cn (S.Z.); 202021200019@mail.bnu.edu.cn (R.W.); huangc_2019@163.com (C.H.); 202021200053@mail.bnu.edu.cn (L.Z.); 2 Institute of Animal Husbandry and Veterinary Medicine, Beijing Academy of Agriculture and Forestry Sciences, Beijing 100193, China

**Keywords:** aneuploidy, dosage compensation, inverse dosage effect, transcription factors, inverse regulators

## Abstract

Aneuploidy, which disrupts the genetic balance due to partial genome dosage changes, is usually more detrimental than euploidy variation. To investigate the modulation of gene expression in aneuploidy, we analyzed the transcriptome sequencing data of autosomal and sex chromosome trisomy in *Drosophila*. The results showed that most genes on the varied chromosome (cis) present dosage compensation, while the remainder of the genome (trans) produce widespread inverse dosage effects. Some altered functions and pathways were identified as the common characteristics of aneuploidy, and several possible regulatory genes were screened for an inverse dosage effect. Furthermore, we demonstrated that dosage changes of inverse regulator *Inr-a*/*pcf11* can produce a genome-wide inverse dosage effect. All these findings suggest that the mechanism of genomic imbalance is related to the changes in the stoichiometric relationships of macromolecular complex members that affect the overall function. These studies may deepen the understanding of gene expression regulatory mechanisms.

## 1. Introduction

The regulatory systems for gene expression of the diploid genomes are quite complicated and balanced. Variations that deviate from balance, such as duplication or loss of a certain chromosome segment or the entire chromosome, known as aneuploidy, disrupt the regulation of the genome [1]. Aneuploidy variation is found to be much more detrimental than euploidy variation in different biological taxa, which is called genetic imbalance [2,3,4,5,6,7]. In the early days of the development of molecular genetics, its basis was thought to be the direct gene-dosage effect of the genes that changed their copies [8,9]. However, many subsequent studies found that quite a few genes on the varied chromosomes produce a similar number of products compared with the normal diploid, that is, dosage compensation occurred [10,11,12]. In addition, some studies proved the expression of genes on the unvaried chromosomes in aneuploidy also have expression changes, and the dominant effect is an inverse correlation with the dosage of the varied region, which is called the inverse-dosage effect [10,11,13,14,15]. According to these findings, dosage compensation is interpreted as the result of the positive dosage effect of genes located on the varied chromosomes being counteracted by the simultaneous inverse-dosage effect of aneuploidy [11,14,15,16,17,18].

By studying the regulators of *white* eye color reporter genes in *Drosophila*, it was found that the regulatory mechanisms responsible for these dosage influences could be reduced to the role of single genes [12,19,20,21]. A lengthy screening process identified 47 dosage-dependent modifiers whose deletion or replication could mimic aneuploidy and influence the expression of the alleles of *white*, of which most had a negative or inverse effect, while a small number had a positive effect [22]. The molecular functions of these modifiers are mainly transcription factors (TFs), signal transduction components and chromatin proteins [22]. Among them, the earliest found and most thoroughly studied inversely acting single gene is *inverse regulator-a* (*Inr-a*, synonymous with *pcf11*). This gene encodes a pre-mRNA cleavage complex II protein, which is involved in the initiation, elongation and termination reactions of transcription, and it produces a nearly perfect inverse effect on the *white* eye color phenotype in *Drosophila* [12,20,21].

The common feature of these dosage-sensitivity modifiers is that they all act in macromolecular complexes. Similar phenomena have been found in other species, for example, abnormal copy number variation (CNV) of transcription factors and signal transduction components in human are usually associated with disease [23,24,25]. A heterozygous gene knockout study in yeast shows that the involvement of macromolecular complex is negatively correlated with fitness [26]. In the aspect of evolutionary genomics, during the deletion of duplicated genes following whole-genome duplications (WGD), those genes involved in macromolecular structural members and interactions tend to be retained for a relatively longer period [27,28,29,30]. The loss of one gene in the duplicate pairs is similar to aneuploidy effects and results in negative fitness, which is usually selected against [3,29,30]. By contrast, in segmental duplications on a small scale, the same classes of genes are often underrepresented [28,29,30,31]. These observations led to a synthetical hypothesis, the Gene Balance Hypothesis (GBH), to explain the mechanism of genomic imbalance [3,28,29].

The gene balance hypothesis suggests that changes in the stoichiometric relationships of macromolecular complex members will affect the assembly kinetics of the complex and ultimately influence the whole function [3,5,32]. This hypothesis has been modeled in some simple scenarios, although the reality may be more complex [32,33,34,35]. The large number of *white* regulators and the similarity to quantitative trait genetics suggest that any individual trait may be influenced by multiple regulatory genes [3,28,36]. Similarly, the increase or decrease of a specific chromosome segment or the changes of a key regulator may affect a variety of biological functions and characteristics [3,28,36]. The explanation is that these transcription factors and signaling components influence downstream regulators in a cascade of interactions, forming a complex hierarchical regulatory network driven by dosage-sensitive regulators [12,22,36].

Aneuploidy often seriously harms the growth and development of organisms [37,38], especially in animals, and is fatal in some cases, so it is more difficult to study the gene expression of aneuploidy. Due to the unique developmental characteristics and the superiority of chromosome aberration strains in *Drosophila melanogaster*, it opens up the possibility of studying aneuploid genomes. In this study, we analyzed two transcriptome high-throughput sequencing datasets of *Drosophila* aneuploidy (including autosomal aneuploidy and sex chromosome aneuploidy) from the GEO database [14,15], and investigated the gene expression characteristics of aneuploidy and the main biological functions of differentially expressed genes. We sought to identify the regulatory genes of aneuploid inverse dosage effects to uncover some key regulators in the hierarchical regulatory network of genomic imbalance. In addition, we also investigated the regulatory role of the classical inverse regulator *Inr-a* and explored its similarity to aneuploidy effects.

## 2. Material and Methods

### 2.1. Drosophila Stocks and Culture

The wild type and *Inr-a* duplication *Drosophila* used for RNA sequencing were cultured in our laboratory. All flies were cultured on cornmeal sucrose medium at 25 °C. The wild type samples were the third instar larva males collected from Canton S stock. The *Inr-a* duplication samples were obtained from crosses of *w[a]; Pcf11Dup/CyO GFP* males with Canton S females, and the progeny larvae without green fluorescence were selected. The *Inr-a* duplication *Drosophila* strain was constructed in a previous study [21].

### 2.2. RNA Extraction and Sequencing

There were two biological replicates in the control group and the *Inr-a* duplication group respectively, with 30 larvae in each replicate. RNA was extracted using TRIzol Reagent from Invitrogen (Thermo Fisher Scientific, Waltham, MA, USA) and processed using *DNase I*, *RNase-Free kit* from Thermo Scientific (Thermo Fisher Scientific, USA). The RNA sequencing was performed as described previously [39]. Briefly, quality detection and concentration measurement were performed on RNA samples, then RNA-Seq library was constructed. The sequencing was performed using Illumina HiSeq X-ten sequencers with the paired-end 150 bp protocol.

### 2.3. RNA Sequencing Analyses

The *Inr-a* data was from the output of Illumina RNA sequencing above. The trisomy 2L and metafemale data were obtained from the GEO database (GSE46354 and GSE41679). All raw data were trimmed and filtered using Trimmomatic (version 0.39) [40] to remove the adaptors and low-quality sequences. FastQC (version 0.11.9) was used to evaluate the quality of the data. Subsequently, transcripts were quantified using Salmon (version 1.4.0) [41]. To be specific, the *Drosophila* reference genome, reference transcriptome, and annotation (version 102) were downloaded from the Ensembl database (http://www.ensembl.org, accessed on 4 January 2021). An augmented hybrid fasta file was created using SalmonTools script. The quasi-type indexes were built with the parameter k-mers = 25 in aneuploidy data and k-mers = 31 in *Inr-a* data, due to the shorter length of reads in aneuploidy data. The transcripts were then quantified directly.

### 2.4. Differential Expression Analysis

Data was consolidated using tximport (version 1.18.0) [42] and imported into DESeq2 (version 1.30.1) [43]. Low expression transcripts were filtered out and differential expression analysis was performed using the parameter fitType = ‘local’. The differentially expressed genes were screened using the standard of padj < 0.05 and fold change >1.25 or <0.8 in the trisomy 2L and metafemale dataset for up- or down-regulation respectively. The density of significantly up-regulated or down-regulated genes along chromosomes were drawn using the R package RIdeogram (version 0.2.2) [44]. The heatmaps were plotted using ComplexHeatmap (version 2.6.2) [45,46].

### 2.5. Enrichment Analysis

Gene Ontology (GO) enrichment analysis, KEGG Pathway enrichment analysis and Gene Set Enrichment Analysis (GSEA) were performed using clusterProfiler (version 3.18.1) [47]. The GO annotations were provided by org.Dm.eg.db (version 3.12.0) and the KEGG database were obtained from online data (https://www.kegg.jp/, accessed on 22 April 2021) [48]. GSEA analysis used empirical Bayes shrunken log2 fold changes data with “apeglm” [49] method in DESeq2.

### 2.6. Protein–Protein Interaction (PPI) Network

PPI network analysis was conducted using STRING 11.0 (https://string-db.org/, accessed on 22 April 2021) [50]. Genes that are up-regulated or down-regulated in all trisomy groups were submitted to the online tool STRING to generate interaction networks. The networks were then imported into Cytoscape (version 3.7.1) [51]. The cytoHubba [52] plugin was used to obtain the degree of each node, and the MCODE [53] plugin was used to cluster the network with the default parameters.

### 2.7. Transcription Factor Analysis

Transcription factor enrichment analysis was performed using RcisTarget (version 1.10.0) [54] in R. The over-expressed TF-binding motifs of the upstream 5kb sequence of the selected down-regulated genes were analyzed using the database it provided. Then the possible regulatory transcription factors were searched based on these enriched motifs and screened by their expressions. The circular diagram of regulatory networks for candidate transcription factors were plotted using Circos (version 0.69.8) [55].

### 2.8. Ratio Distribution

The data from RNA sequencing was normalized to calculate CPM (counts per million). The ratio of the experimental group to the control group was averaged across biological replicates, and then the frequency distributions were plotted in bins of 0.05 using ggplot2 (version 3.3.3) [56]. The microarray data used in the ratio distribution plots and boxplots came from the GEO database (GSE53010 and GSE36736) without logarithm conversion.

### 2.9. Relative Quantitative PCR

The rest of the RNA produced in sequencing was used for RT-PCR. cDNA was synthesized using the *TransScript* one-step gDNA Removal and cDNA Synthesis SuperMix. The real-time PCR was performed using *TransScript* Tip Green qPCR SuperMix (+Dye II) with the primers listed in Table S1. The housekeeping gene *β-tubulin* served as an internal control. 2^−ΔΔCt^ method was used for relative quantitative calculation.

### 2.10. Data Availability

The sequencing data of *Inr-a* duplication *Drosophila* has been deposited in the Gene Expression Omnibus (GEO) database (https://www.ncbi.nlm.nih.gov/geo/, accession no. GSE180089) and the Sequence Read Archive (SRA) (https://www.ncbi.nlm.nih.gov/sra, accession no. SRP328302). The public data used in this study were downloaded from GSE46354, GSE41679, GSE53010 and GSE36736 (accessed on 4 January 2021). The public datasets GSE46354 and GSE41679 include the high-throughput transcriptome sequencing data of trisomies of the left arm of chromosome 2 (2L) and of the X chromosome in *Drosophila* respectively. The other two public datasets contain the expression microarray data of the overexpression or mutation of transcription factor genes in *Drosophila* respectively.

## 3. Results

### 3.1. Identification of Differentially Expressed Genes and Dosage Compensation

To identify the differentially expressed genes (DEGs) in aneuploid *Drosophila*, we downloaded the sequencing data from the public datasets GSE46354 and GSE41679, which respectively includes the RNA-seq data of trisomies of 2L and of the X chromosome in *Drosophila*. After downloading the raw data, Salmon was used for transcriptome quantitation, and R package DESeq2 was used for differential expression analysis. Since the complete dosage effect in trisomy tends to upregulate the genome approximately 1.5-fold in theory, DEGs were defined as adjusted *p*-value < 0.05 and fold change > 1.25 or < 0.8.

**Figure 1:**
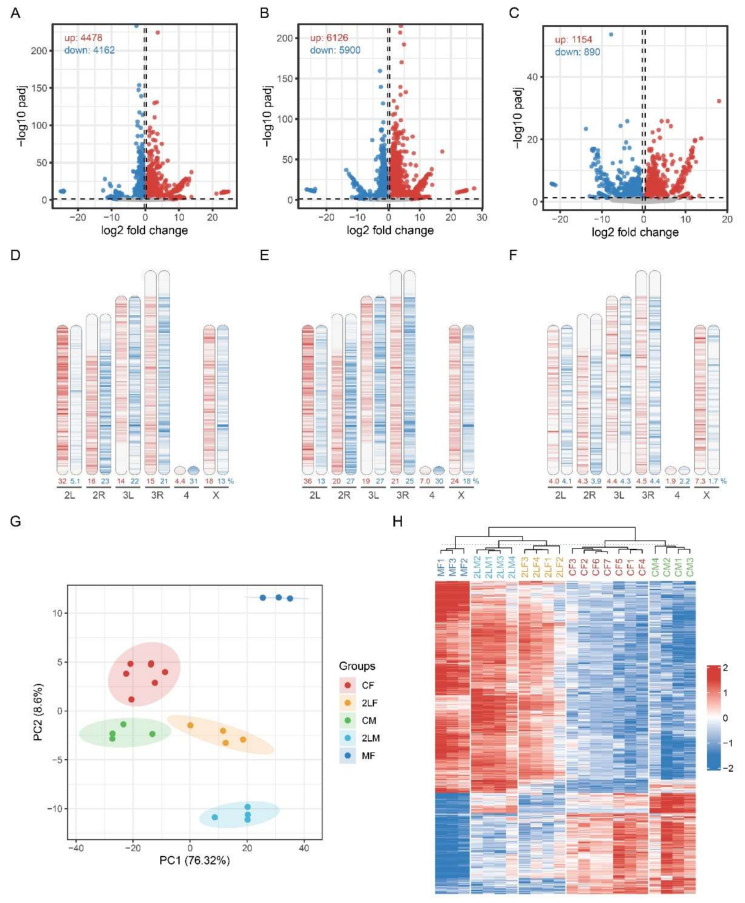
Overview of the differentially expressed genes (DEGs) in three kinds of aneuploidy. (**A**–**C**) Volcano plots of DEGs in trisomy 2L female (**A**), trisomy 2L male (**B**) and metafemale (**C**) compared with normal diploid of the corresponding sex. DEGs are defined as adjusted *p*-value < 0.05 and fold change >1.25 or <0.8. (**D**–**F**) Density heatmaps of the distribution of DEGs along *Drosophila* chromosomes in trisomy 2L female (**D**), trisomy 2L male (**E**) and metafemale (**F**). The red bands represent up-regulated genes, and the blue bands represent down-regulated genes. The darker colors show a greater density of DEGs. The number below the chromosomes indicates the percentage of up- or down-regulated genes in the total number of genes in this chromosome. (**G**) Principal component analysis (PCA) plot of all aneuploidy samples and control groups. The circles represent 95% confidence intervals. (**H**) Heatmap of all aneuploidy samples and control groups. CF, wildtype female control; CM, wildtype male control; 2LF, trisomy 2L female; 2LM, trisomy 2L male; MF, metafemale.

We found a large number of DEGs in trisomy compared with the normal diploid, especially in trisomy 2L more than in metafemales (Figure 1A–C). By observing the distribution of DEGs, we found that the up-regulated and down-regulated genes were widely distributed on each chromosome (Figure 1D–F), suggesting that aneuploidy has a wide range of effects on gene expression in the whole genome. Based on the density heatmaps of the DEGs, 30% of genes located on chromosome 2L exhibit up-regulation, while the expression of the remaining genes is not significantly up-regulated, indicating that dosage compensation occurs in 2L (Figure 1D,E). Gene expression changes also occur on autosomes other than 2L and the sex chromosomes, with both up-regulation and down-regulation. More down-regulated genes are found, indicating that aneuploidy modulates the gene expression not only on the varied chromosomes, but also on other chromosomes in trans. These modulations could be positively or negatively correlated with the changed chromosome dosage, but inverse correlations are the most common, which is an inverse dosage effect. And we found that the position distribution of DEGs in trisomy 2L female and male is very similar, suggesting that the regulation is not random, but specific. Furthermore, dosage compensation on the X chromosome and an inverse dosage effect on the autosomes can also be observed in metafemales (Figure 1F).

### 3.2. Common Differentially Expressed Genes and Enrichment Analysis

GO function and KEGG pathway enrichment analysis were performed for DEGs in three kinds of aneuploidy, respectively. We found that there are a large number of the same GO terms in biological process (Figure S1A). The commonly enriched functions include immune system progress, response to biotic stimulus, positive regulation of cell communication, cell morphogenesis involved in differentiation, post-embryonic animal morphogenesis and organ development, organic acids and carbohydrate metabolic process and so on, indicating that the influences are involved in immune system, cellular communication, differentiation and development, metabolic and other important processes, which is consistent with the phenomenon that aneuploidy affects the growth and development of organisms [37,38]. The enrichment analysis of molecular function and cellular component showed that the DEGs in two autosomal trisomies are enriched in more identical GO terms, while the DEGs in the triple X metafemale are enriched in different terms (Figure S1B,C). The significantly enriched pathways in aneuploidy *Drosophila* include glucose metabolism, lipid metabolism, lysosome, and so on (Figure S1D).

**Figure 2:**
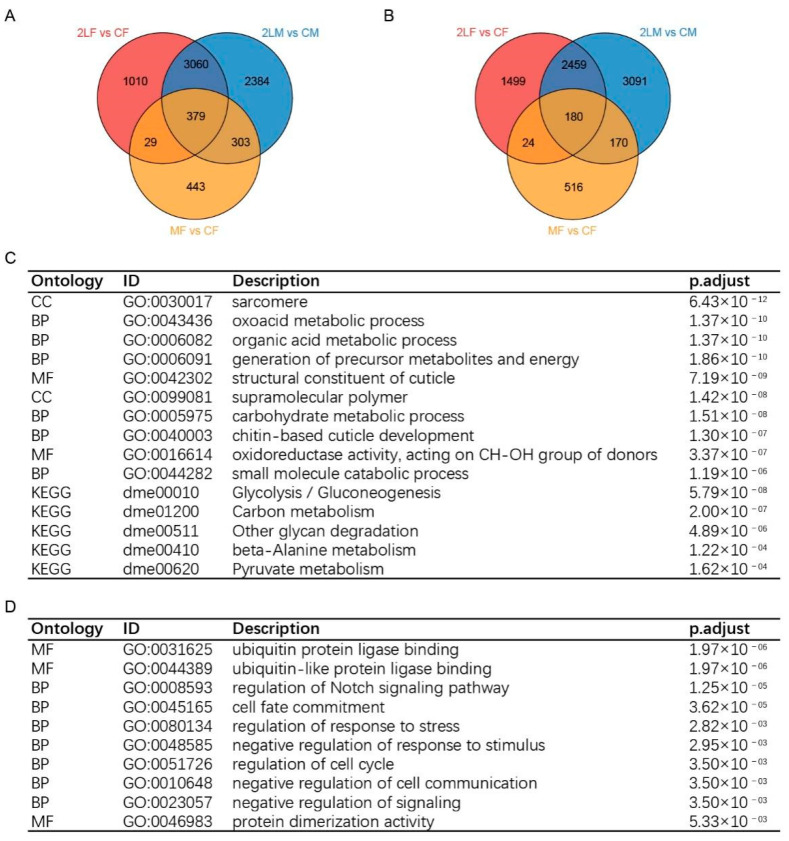
Common up-regulated or down-regulated differentially expressed genes in three kinds of aneuploidy. (**A**,**B**) Venn diagrams show the number of up-regulated (**A**) or down-regulated (**B**) DEGs in each comparison. CF, wildtype female control; CM, wildtype male control; 2LF, trisomy 2L female; 2LM, trisomy 2L male; MF, metafemale. (**C**) Top 10 enriched GO terms and top 5 enriched pathways of common up-regulated DEGs in all trisomies. (**D**) Top 10 enriched GO terms of common down-regulated DEGs in all trisomies.

By obtaining the intersection of the up-regulated or down-regulated genes of three kinds of aneuploidy, 379 common up-regulated genes and 180 common down-regulated genes were found (Figure 2A,B). The principal component analysis (PCA) plot shows significant differences among each genotype, and the heatmap clearly separates the trisomy from the normal diploid (Figure 1G,H). To analyze the functions of these genes, we found that the up-regulated functions and pathways in aneuploidy were mainly concentrated in small molecule metabolism and generation of energy, supramolecular polymer, structural constituent of cuticle, sarcomere, etc. The functions of down-regulated genes include negative regulation of cell signaling, cell cycle regulation, response to stress, cell fate commitment, ubiquitin protein ligase binding, Notch signaling pathway, etc. (Figure 2C,D). Therefore, in aneuploidy, the genes affected by a dosage effect are mainly those related to the basic life activities of the cell, while the genes affected by inverse dosage effect are mostly those that play a crucial regulatory role in the regulation of signaling pathway, cell proliferation and differentiation, protein degradation and so on.

### 3.3. Protein Interaction Analysis of Common Differentially Expressed Genes

**Figure 3:**
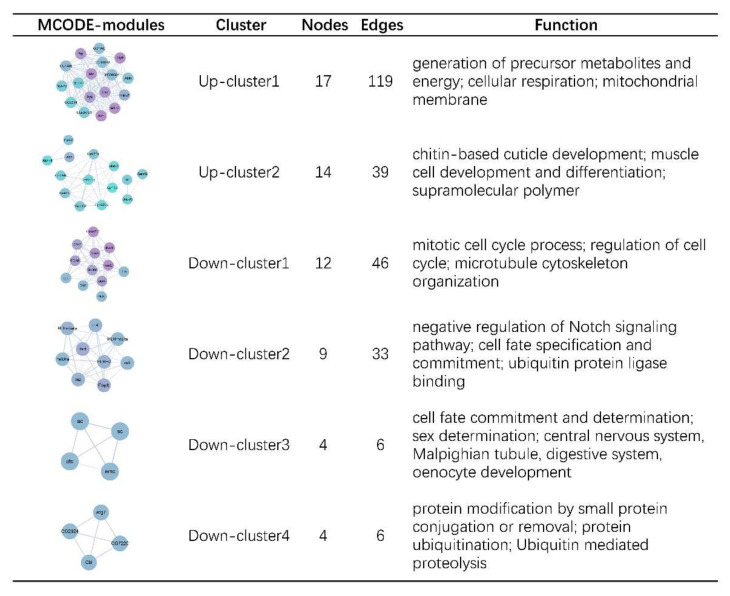
The MCODE-modules of protein-protein interaction (PPI) networks of common up- or down-regulated DEGs. The top two up-regulated modules and top four down-regulated modules with unique functions are displayed. The number of nodes and edges, and the significant functions for each module are listed. The purple nodes have a higher degree of connection in the whole networks.

Using the online website String to analyze the protein interactions of the up- or down-regulated genes in aneuploidy, we found that the protein-protein interaction (PPI) network of commonly up-regulated genes contained 197 nodes and 694 edges (Figure S2A), which is large and complex, while the relatively sparse PPI network of common down-regulated genes contained 94 nodes and 209 edges (Figure S2B). The top 20 genes with the highest degrees in each network are listed, suggesting that they might play an important role in the overall regulatory network (Figure S2C,D). Subsequently, MCODE analysis was used to identify 10 clusters in the PPI network of common up-regulated genes, and the top two up-regulated clusters with the highest scores were displayed (Figure 3). The first and largest cluster contains 17 genes, whose functions are mainly involved in the generation of precursor metabolites and energy, cellular respiration and mitochondrial membrane. The second cluster contains 14 genes, whose functions are involved in the development of chitin-based cuticle development, muscle cell development and differentiation, and the formation of supramolecular polymers (Figure 3). The remaining eight up-regulated clusters functionally are similar to these top two clusters and are all involved in metabolic or supramolecular complexes. Five clusters have been identified in the PPI network of common down-regulated genes, with different functions, and the top four are displayed in Figure 3. The largest down-regulated cluster includes 12 genes, in charge of mitotic cell cycle process, regulation of cell cycle and microtubule cytoskeleton organization, showing the effect of aneuploidy on normal cell division cycle. The second down-regulated cluster is mainly associated with Notch signaling pathway, cell fate specification and commitment and ubiquitin protein ligase binding. The third down-regulated cluster is involved in a variety of developmental processes, including sex determination and the development of central nervous system, Malpighian tubule, digestive system and oenocyte. The fourth down-regulated cluster mainly involves ubiquitin mediated proteolysis. Therefore, down-regulated genes in clusters 2, 3, and 4 play an important regulatory role in cell life activities, and may act as hub genes to regulate the growth and development in aneuploidy.

### 3.4. Transcription Factor Analysis of Inverse Dosage Effect Genes

Indeed, it has been found that the influence of aneuploidy can be reduced to the action of single genes in *Drosophila* and the genes respond to dosage effects of aneuploidy usually include transcription factors (TFs), signal transduction components, and chromatin proteins [22]. The inverse dosage effect operates at the transcriptional level, and the promoter region plays a key role in this effect [12,17]. Therefore, to search for regulatory TFs in aneuploidy, we sought from the genes affected by inverse dosage effect. By enrichment analysis of TFs-binding motifs in the upstream 5 kb sequences of the common down-regulated DEGs in the trisomy, regulatory transcription factors with high confidence were selected through these over-represented motifs. We found 36 enriched transcription factors, among which the top 10 and their binding motif with the highest score were listed in Figure S3A. By comparing the expression of these transcription factors in trisomies and normal diploids, we found that *ac* was significantly down-regulated in all trisomies while *kay* was significantly up-regulated (Table 1; Figure S4A,B), suggesting that they may be key transcription factors that play a regulatory role in inverse dosage effect. They are modulated by aneuploidy, so something else affects them, but they could be involved in a cascade.

**Figure 4:**
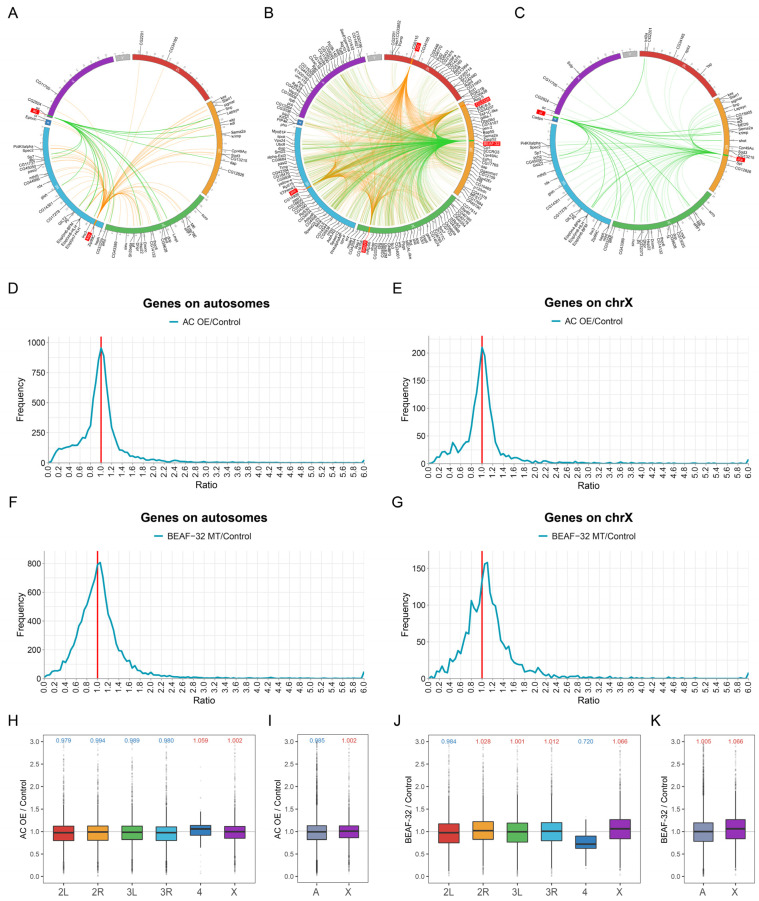
Modulation of gene expression by candidate transcription factors. (**A**–**C**) The circular plots show the target gene networks of candidate TFs in all trisomies (**A**), trisomy 2L females and males (**B**) and trisomy females (**C**). The green lines indicate that the expression of this TF is down-regulated in trisomies, and the yellow lines indicate that the expression of this TF is up-regulated in trisomies. Candidate TFs are labeled with red rectangles. Target genes are partially listed on the periphery of the chromosomes. (**D**,**E**) Ratio distributions of gene expression in TF *ac* overexpression *Drosophila* compared with control group. (**F**,**G**) Ratio distributions in TF *BEAF-32* mutation *Drosophila* compared with control group. Genes are divided into autosomes (**D**,**F**) and X chromosome (**E**,**G**) according to their positions. The X-axis indicates the ratio of expression, and the Y-axis indicates the frequency of the ratios that fall into each bin of 0.05. The red solid line represents the ratio of 1.00 which means no change in gene expression. (**H**,**I**) Boxplots of ratios in *ac* overexpression *Drosophila* compared with control group. Genes are divided into each chromosome (**H**) or autosome and sex chromosome (**I**) according to their positions. (**J**,**K**) Boxplots of ratios in *BEAF-32* mutation *Drosophila* compared with control group. Genes are divided into each chromosome (**J**) or autosome and sex chromosome (**K**) according to their positions. The gray solid line represents the ratio of 1.00 which means no change in gene expression. The numbers at the top of the boxplots indicate the median, blue represents down-regulation and red represents up-regulation.

In addition, considering the differences in aneuploidy for the autosomes and the sex chromosome, as well as the differences between male and female, we also analyzed the genes that are affected by an inverse dosage effect in both trisomy 2L females and males, and in both trisomy 2L females and triple X metafemales (Figure S3B,C). Enriched transcription factors differentially expressed in both aneuploidies were identified as autosomal aneuploidy and female sexual specific regulators, respectively (Figure S4C–I). Nine transcription factors were finally screened out, and their information was listed in Table 1. The key transcription factors obtained from the three screening groups form a complex regulatory network with the predicted inverse dosage effect genes they regulate, where a single transcription factor regulates a large number of genes on different chromosomes (Figure 4A–C).

### 3.5. Validation of Candidate Transcription Factors in Public Database

To analyze whether the key transcription factors we identified act like inverse dosage regulators, that is, whether the overexpression or mutation of a single gene causes genome-wide inverse dosage effect similar to aneuploidy, we explored two related datasets from the GEO public database. The dataset GSE53010 includes expression microarray data of the overexpressed transcription factor *ac* in the notum of *Drosophila*, and the dataset GSE36736 includes microarray data of the mutation of *BEAF-32* gene in wing imaginal tissue of *Drosophila*. The ratio distribution can provide a whole genome-wide landscape of changes in gene expression, especially the subtle changes close to the control group, without being limited by the statistical power of small sample size [18]. The mean value of gene expression of mutant or overexpressed *Drosophila* were compared with controls, and these ratios were plotted with bins of 0.05. The *ac* gene is located on the X chromosome and we found that the major peaks are all located at the ratio of 1.00, which was drawn according to the autosome and sex chromosome, respectively (Figure 4D,E), suggesting that the expression of most genes did not change significantly. However, an obvious tailing is observed on the left of the main peak, but not on the right side, suggesting that the expression of some genes was decreased in *ac* overexpressed *Drosophila*. There is a shoulder peak at the ratio of 0.7 (Figure 4D,E), similar to the inverse dosage effect found in trisomy. And there is a minor peak at the ratio of 0.45 (Figure 4E), approaching to the double inverse dosage effect (2/3 × 2/3), which has also been previously reported [14]. At the same time, we also plotted the boxplots of gene expression ratio of each chromosome (Figure 4H,I). The median of gene expression ratios on four major autosomal arms are all lower than 1.0, while the median ratio on the X chromosome is slightly higher than 1.0, proving that the overexpression of *ac* on the X chromosome produced trans inverse dosage effect on the autosome. Different from other autosomes, the median ratio of chromosome 4 increases significantly, potentially due to its different evolutionary pathways and heterochromatin composition.

In the ratio distribution of mutation of *BEAF-32* on the chromosome 2R, the major peak shifts to the right as a whole centered at the ratio of 1.05–1.10 and is stronger on the X chromosome (Figure 4F,G), indicating that *BEAF-32* mutation leads to slight up-regulation of the overall gene expression. There is a shoulder peak near the ratio of 1.2 and 1.5 (Figure 4F,G), which represents a different level of up-regulation. In the boxplot, the median of gene expression ratio on most autosomes is higher than 1.0, and the increase on the X chromosome is even more pronounced, demonstrating the inverse dosage effect of *BEAF-32* mutations on the autosomes (Figure 4J,K). The median ratio of chromosome 4 decreases significantly, also different from other autosomes. Thus, gene expression tends to be down-regulated in *ac* overexpressed *Drosophila*, while up-regulated in *BEAF-32* mutation. It shows that the expression changes of two transcription factors we selected exert negative effects on a subset of the genome, similar to the previously described inverse regulators.

### 3.6. Inverse Regulator Inr-a/pcf11

**Figure 5:**
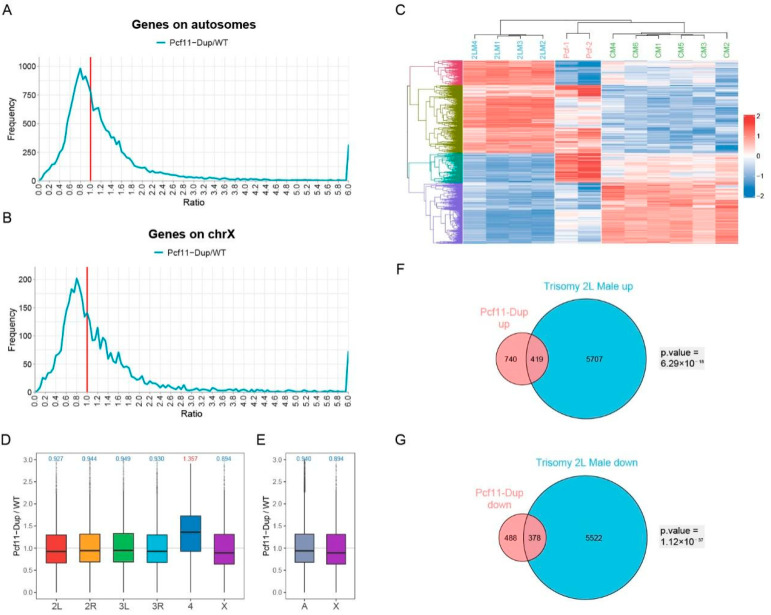
Effect of inverse regulator *Inr-a/pcf11* on global gene expression. (**A**,**B**) Ratio distributions of *Inr-a* duplication male compared with wildtype male. Genes are divided into autosomes (**A**) and X chromosome (**B**) according to their positions. The X-axis indicates the ratio of expression, and the Y-axis indicates the frequency of the ratios that fall into each bin of 0.05. The red solid line represents the ratio of 1.00 which means no change in gene expression. (**C**) Heatmap of common differentially expressed genes in *Inr-a* duplication male and trisomy 2L male compared with wildtype male. CM, wildtype male control; 2LM, trisomy 2L male; Pcf, *Inr-a* duplication male. (**D**,**E**) Boxplots of ratios in *Inr-a* duplication male compared with wildtype male. Genes are divided into each chromosome (**D**) or autosome and sex chromosome (**E**) according to their positions. The numbers at the top of the boxplots indicate the median, blue represents down-regulation and red represents up-regulation. (**F**,**G**) Venn diagrams show the number of up-regulated (**F**) or down-regulated (**G**) genes in *Inr-a* duplication male and trisomy 2L male. DEGs are defined as adjusted *p*-value < 0.05 and fold change >1.25 or <0.8 in trisomy 2L male, and adjusted *p*-value < 0.05 in *Inr-a* duplication male. Fisher’s exact test *p* values are shown beside the Venn diagrams.

The first trans-acting regulatory gene found in *Drosophila* was named *Inverse regulator-a* (*Inr-a*), synonymous with *pcf11* [20,21]. The study found that the heterozygote of *Inr-a* mutation increased the expression of *white* approximately about twofold, while the increase to three copies of the gene reduced the expression of *white* by about two-thirds [20,21]. However, the inverse regulation of the classical inverse regulator *Inr-a* has only been studied on a few genes, rather than explored on a genome-wide scale. Therefore, to explore whether there is similarity between the *Inr-a* mutation and trisomy, we performed RNA-sequencing and analyzed the transcriptome of *Drosophila* larvae with *Inr-a* duplicates on chromosome 2R and compared this three-copies of *Inr-a Drosophila* with autosomal trisomy. Several randomly selected genes were used for RT-qPCR to verify the sequencing results (Tables S1 and S2). According to the gene expression ratio distribution plots of the autosomes and the X chromosome in *Drosophila* with an *Inr-a* duplication, the positions of the major peaks are all lower than 1.0, and locate at 0.8 (Figure 5A,B). These results indicate that the expression levels of a large number of genes in the *Inr-a* duplicated *Drosophila* are down-regulated, which is similar to inverse dosage effect in trisomy, but the down-regulated magnitude is less than 2/3, which might because the inverse dosage effect caused by the duplication of a single gene is not as strong as the whole chromosome arm.

There is a shoulder peak near the ratio of 1.0 (Figure 5A,B), indicating that the expression of some genes was not affected by the *Inr-a* duplication. In the corresponding boxplots, we can also observe the same trend that, with the exception of up-regulated median gene expression ratio on chromosome 4, the median gene expression ratio on all other chromosomes is down-regulated (Figure 5D,E). The clustering heatmap of the common DEGs of the *Inr-a* duplication males and trisomy 2L males shows a high degree of consistency within each genotype (Figure 5C). These genes are divided into four groups by unsupervised clustering. The majority of genes are commonly up-regulated or down-regulated in both *Inr-a* and trisomy 2L, and less than half of the genes are regulated in different directions in *Inr-a* and trisomy 2L, respectively. The genes significantly up-regulated in *Inr-a* duplication are significantly enriched in the up-regulated genes of trisomy 2L (Fisher’s exact test *p*-value = 6.29 × 10^−18^) (Figure 5F), and the genes significantly down-regulated in *Inr-a* duplication are also significantly enriched in the down-regulated genes of trisomy 2L (Fisher’s exact test *p*-value = 1.12 × 10^−37^) (Figure 5G), demonstrating that the regulation of gene expression caused by *Inr-a* duplication is similar to the aneuploidy effect caused by trisomy. Thus, single dosage-sensitive regulator, *Inr-a*, has an extensive inverse dosage effect on a subset of the genome, not limited on the *white* locus, indicating that the function of the trans-acting single genes is universal.

## 4. Discussion

Aneuploidy variation can seriously affect the development and viability of organisms and is usually more harmful than euploidy variation. This phenomenon and its mechanism are summarized as the Gene Balance Hypothesis [3,28,29,37,38,57]. Many studies have found that the addition or deletion of a large chromosome segment or entire chromosome in the karyotype can lead to genomic imbalance and affect gene expression across the genome, not only the varied chromosome. This effect is usually inversely related to the changes of chromosome numbers, and is known as the inverse dosage effect [5,6,7]. Several recent studies have examined the inverse dosage effect of aneuploidy in a variety of species in detail, such as maize, Arabidopsis and fruit flies [6,7,14,15,58,59]. These studies used several different transcriptome analysis methods all to avoid the artifacts of normalizing the measurements of varied chromosomes to the remaining parts of the genome [12,18], and were further validated at the phenotypic or absolute expression levels. In our analysis of autosomal and sex chromosome aneuploidy *Drosophila*, inverse dosage effect and dosage compensation are also observed through the distribution of differentially expressed genes on chromosomes (Figure 1D–F), which is consistent with the analysis of ratio distributions in previous studies [14,15].

The prevalence of inverse dosage effect in aneuploidy provides a possible explanation for the mechanism of dosage compensation. As early as forty years ago, studies on the subdivision of aneuploid chromosome found that dosage changes in a small region surrounding structural genes would exhibit direct gene dosage effect, while dosage changes in another large region on the same chromosome would produce inverse regulation [16,17]. The combination of these observations suggests that dosage compensation occurs because the genes located on the varied chromosomes are regulated by both structural gene dosage effect and inverse dosage effect, and these two reactions cancel each other [3,5,11].

The classic case of dosage compensation is the heterogamous individuals in the organisms whose sex determination rely on sex chromosomes. For example, the single X chromosome in male *Drosophila* is over-activated in order to balance the double dosage in females. Early studies have suggested that there are male-specific lethal (MSL) complexes specifically enriched on the X chromosome in males, which mediates the two-fold activation of the X chromosome [12,60]. MSL2, the subunit of MSL complex, mediates the assembly of the whole complex and does not exist in females due to the inhibition by SXL (Sex Lethal). The subunit MOF specifically acetylates histone H4K16, which is considered to be a marker of transcriptional activation [61,62]. Evidence that the MSL complex directly causes dosage compensation in *Drosophila*, such as male-specific lethal gene mutations reduce the expression of X chromosome relative to autosomal expression, assume that autosomal expression remains unchanged [63,64], possibly causing artifacts. However, when the absolute expression level or phenotypes were observed later, no loss of X chromosome dosage compensation was observed, but autosomal expression was increased [61,65,66]. In addition, targeting MOF to reporter genes in yeast resulted in an approximately 10-fold increase in expression that was far greater than the regulation of dosage compensation in *Drosophila* [67]. In another study, acetylation levels of the MOF-targeted reporter genes were increased in both male and female *Drosophila*, but the expression was increased only in females, but not in males with a completely assembled MSL complex [39]. This suggests that the MSL complex may cause a constraining activity that counteracts the effect of histone hyperacetylation on gene expression [68].

There is increasing evidence that the effective modulation magnitude of dosage compensation is not achieved through a single mechanism, but through a complicated integration of activation and inhibition [1,36,39]. Considering the universality of inverse dosage effect in aneuploidy, an explanation is proposed. The imbalance of the single X chromosome relative to the rest of the genome in male *Drosophila* results in extensive inverse dosage effects to increase the expression level of the whole genome. Unlike aneuploidy caused by chromosomal abnormalities, sex chromosome monosome is emerged over a long period of evolution, so there must be some mechanism that inhibits the harm of genomic imbalance caused by aneuploidy. To avoid autosomal activation in trans, the MSL complex may have evolved the role of isolating histone modifiers such as MOF on the X chromosome [12,14,15,36,39]. As the Y chromosome degenerated and the hemizygous region expanded, the X chromosome accumulated more and more histone modifiers than it needs to compensate. Therefore, an inhibitory activity associated with the MSL complex evolved to prevent overexpression of the X chromosome and provide the appropriate level of regulation to achieve dosage compensation [12,14,15,36,39].

In various studies, common characteristics of aneuploidy driven by genomic imbalance have been identified, such as altered metabolism, lower viability, genomic instability, and altered proteostasis [57,69,70]. In this study, we found that a number of metabolic processes in *Drosophila* are affected by aneuploidy states, such as organic acid and carbohydrate metabolic process. Most of these metabolic functions are significantly enriched in up-regulated DEGs (Figure 2D; Figure S1), which is consistent with previous reports that aneuploidy requires more carbohydrates or energy [57,70]. It is suggested that metabolic disorder may be the direct physiological cause of aneuploidy variation that harms the survival of cells or organisms. In addition, we observed that cell cycle regulation, mitosis and other related terms are significantly enriched in down-regulated DEGs, and form the largest down-regulated cluster (Figure 2D and Figure 3). The abnormal number of chromosomes will affect the division and proliferation of cells, which is expected, since the normal separation of chromosomes depends on the symmetry of two copies. This effect has also been demonstrated in other studies, such as G1 delay in aneuploidy yeast and proliferation defects of mammalian aneuploidy cells [57,70,71].

Aneuploidy is a common feature of cancer, with data showing that more than 90% of solid tumors in humans contain aneuploidy [72,73]. Although the causal relationship between aneuploidy and tumors is uncertain at present, studies have found that aneuploid cells with genomic instability may even exhibit the ability to proliferate indefinitely, similar to cancerous cells [70,74,75]. A recent study found that induction of Notch signaling in polyploid cells leads to malignant proliferation, loss of cell polarity, and further development into tumors with high genomic instability [76]. Interestingly, our study shows that genes associated with the Notch signaling pathway are abnormally expressed in aneuploidy *Drosophila* (Figure 2D and Figure 3), suggesting a close association between aneuploidy and tumor formation. We also found that aneuploidy states affect cell fate commitment and determination, and influence the development of multiple organs and systems (Figure 2D and Figure 3), which is consistent with the fact that aneuploidy variation usually severely affects the growth and development of organisms [37,38]. Protein stability in aneuploidy cells is also affected, possibly because aneuploidy interferes with protein synthesis and alters sensitivity to protein folding, leading to protein degradation [57,69]. We found that aberrantly expressed genes in *Drosophila* aneuploidy enriched the function of protein ubiquitination (Figure 2D and Figure 3), revealing that aneuploidy may affect protein degradation by regulating the ubiquitination process.

Some details of the regulation of aneuploidy have also been identified. The magnitude of the modulation of gene expression in aneuploidy is positively correlated with the degree of genomic imbalance, that is, the effect of dosage is progressive [7,59]. In general, modulation in aneuploidy is more spread than in a ploidy series [6,59]. The expression level of cis genes usually ranges from dosage compensation to gene dosage effect, and the most common regulation of trans genes is inverse dosage effect [7,58,59]. The regulation mechanism of cis genes affected by dosage compensation and trans genes affected by inverse dosage effect are related, and they share the same molecular basis [58,59]. Genes of different functional classes show different responses to aneuploidy. Transcription factors, signal transduction components, and organelle targeted protein genes are more tightly inversely affected, and transcription factors and their targets showed considerable discordance [6,7,59]. This suggests that altered regulatory stoichiometry is the main cause of genetic imbalance. In addition, evidence from evolutionary genomics, gene copy number variation, and quantitative trait similarity all indicate that stoichiometry of gene regulation plays a potential role in aneuploidy genetic balance [3,22,23,24,25,26,27,28,29,30,31].

The study of *Drosophila* dosage sensitive modifiers for *white* eye color gene phenotypes found that, the effect of aneuploidy on gene expression can be reduced to the action of single genes, which are typically components of macromolecular complexes and multicomponent interactions [12,19,20,21,22]. The first trans-acting modifier *Inr-a* was found to have a precise inverse effect on the expression of the *white* gene [20,21]. In our study, we demonstrated that the dosage changes of inverse regulator *Inr-a* can mimic the effects of aneuploidy across the whole transcriptome. The increase of *Inr-a* copy number could induce a broad inverse dosage effect on gene expression throughout the whole genome, and this effect is similar to the modulation of trisomy 2L, but the magnitude is slightly less (Figure 5). These results indicate that the effect in aneuploidy is the result of a series of dosage-sensitive regulators as members of the macromolecular complexes, which together constitute the complicated hierarchy of gene expression regulation network.

In order to find the key regulatory genes in the genomic imbalanced hierarchical regulatory network, we focused on the typical types of macromolecular complexes and looked for some transcription factors that might contribute to inverse dosage effect in aneuploidy. *ac* (*achaete*) is a basic helix-loop-helix (bHLH) transcription factor, usually formed in complex with *sc* (*scute*), which controls the development of the dorsal plate and setae in *Drosophila* and is thought to be involved in the development of the nervous system and sensory organs [77,78]. In these processes, *ac* interacts with the Notch signaling pathway [79,80]. Through the analysis of gene expression microarray data of *ac* overexpressed *Drosophila* in the public database, we found that the overexpression of *ac* results in down-regulation of many genes (Figure 4D,E), similar to the influence of inverse dosage effect, indicating that *ac* may play an important regulatory role in aneuploidy as a dosage-sensitive regulator. *BEAF-32* (*boundary element-associated factor of 32kD*) is a DNA binding protein that can bind scs boundary element of *hsp70* and hundreds of other sites on *Drosophila* chromosomes [81,82]. Boundary elements or chromatin insulators can isolate chromosomal regions into functionally independent parts by blocking the interactions of promiscuous promoters or enhancers [81,82]. In addition to the function of chromatin organization, *BEAF-32* also regulates cell cycle genes by controlling H3K9 methylation [83]. In the *BEAF-32* mutant *Drosophila*, we found that the gene expression level of a subset of the transcriptome is slightly up-regulated (Figure 4F,G), suggesting that the reduced expression of *BEAF-32* may produce inverse modulation.

Several other potential aneuploidy dosage-sensitive regulatory genes play important roles in a variety of biological processes. *kay*, also known as *Fos* or *AP-1*, is related to *Drosophila* compound eye development, dendrite morphogenesis, embryo development, and cell cycle regulation [84,85,86,87,88]. *Jra* is a homologue of the mammalian transcription factor *Jun*. Both *Jra* and *kay* are basic region-leucine zipper (bZIP) transcription factors and interact with each other [84,89]. *Jra* plays a role in embryonic development, morphogenesis, neuronal development and plasticity in *Drosophila* [89,90,91]. *crp*, also known as *AP-4*, acts on muscle development and cellular branching of the terminal cells at the ends of tracheal tubes [92,93]. Moreover, as a downstream target gene of *Myc*, *crp* can promote the growth of cell and organ, affect cell cycle and survival of organisms [93,94]. The transcription factor *BtbVII* is thought to play a role in embryonic and larval development of *Drosophila*. *CG3328*, expressed in embryonic and larval garland cells, is the orthologous gene of human gene *MYRF* (*myelin regulatory factor*), regulating myelination process [95].

Another possible inverse regulator *pnr* is a GATA factor, which activates the achaete-scute (ac-sc) complex and plays a role in nervous system development [96,97,98]. It was found that *pnr*-driven neural development requires the involvement of chromatin remodeling factor *Iswi* [98,99], and *Iswi* may have the activity of inhibiting gene overexpression in *Drosophila* dosage compensation [12,99]. *ci*, homologous with *Gli*, is an effective transcription factor of Hedgehog (Hh) signal transduction pathway, which is necessary for the pattern formation, morphogenetic and growth control of early development in *Drosophila* [100,101,102]. *ci* can form full-length transcription activator in the presence of *hh* (hedgehog) and truncated repressor in the absence of *hh* [103]. It is worth noting that *hh* is one of the 47 trans-acting modifiers previously identified in *Drosophila* [22], and thus it is speculated that transcription factor *ci* may be a member of *hh* regulatory pathway in aneuploidy.

Besides transcription factors, two other typical macromolecular complexes, signal transduction components and chromatin proteins, are also analyzed [22]. GSEA analysis of the genes contained in the relevant GO terms in aneuploidy revealed that the significant changes included the G-protein-related second messenger signaling pathway, Toll signaling pathway, Notch signaling pathway, etc. In addition, chromatin assembly or disassembly related genes tended to be down-regulated in all three kinds of aneuploidy (Figure S5).

In summary, we analyzed transcriptome sequencing data of autosomal aneuploidy and sex chromosome aneuploidy in *Drosophila* and observed dosage compensation in the varied chromosomes and a genome-wide inverse dosage effect. Based on the characteristics of differentially expressed genes, we found that genomic imbalance affects cell proliferation and differentiation, protein stability, as well as Notch and other signaling pathways, interferes with many physiological metabolic processes, and ultimately influences the growth, development and survival of organisms. In search of the regulatory genes of aneuploid inverse dosage effect, we analyzed the transcription factors and other macromolecular complexes, and predicted nine possible regulators that may play important roles in the regulation network of genomic imbalance. They have indispensable functions in the early development, morphogenesis, cell cycle, and nervous system of *Drosophila*. In addition, we analyzed the expression profiles of two transcription factors, *ac* and *BEAF-32*, mutation or overexpression *Drosophila*, and found that mutation of candidate regulatory genes would lead to the tendency of up-regulation across the genome, while overexpression showed an opposite trend, that is to produce an inverse effect. We also studied a classical inverse modifier, *Inr-a*, whose increase in dosage produces inverse dosage effect across the transcriptome and is similar to the trisomy of chromosome 2 left arm. Therefore, this study highlights the existence of dosage compensation in aneuploidy, which is realized through widespread inverse dosage effects, and the mechanism is that the altered stoichiometric relationship of multi-subunit complex members caused by genomic imbalance ultimately affects the overall function. The candidate aneuploidy regulatory genes and related pathways need to be further studied, and the study of dosage-sensitive regulators will help to further understand the regulation mechanism of gene expression in aneuploidy.

## Figures and Tables

**Table 1:** Transcription factors expression in various aneuploids.

Groups	Enriched TF	TF Location	Function	2LFvsCF	2LMvsCM	MFvsCF
All trisomy	*ac*	X	BHLH transcription factor; interacts antagonistically with the Notch signaling pathway; nervous system development	down	down	down
All trisomy	*kay*	3R	involved in multiple biological processes	up	up	up
Trisomy 2L	*BEAF-32*	2R	chromatin domain insulator function, gene regulation and genome organization	down	down	normal
Trisomy 2L	*BtbVII*	3L	embryonic/larval nervous system; extended germ band embryo	up	up	normal
Trisomy 2L	*CG3328*	2R	embryonic/larval garland cell; orthologous to human *MYRF* (myelin regulatory factor)	up	up	normal
Trisomy 2L	*crp*	2L	controls cellular branching of the terminal cells at the ends of tracheal tubes; a downstream target gene of *Myc*	up	up	normal
Trisomy 2L	*pnr*	3R	an activator of proneural achaete-scute complex genes; dorsal cell fate determination; nervous system development	up	up	normal
Trisomy female	*ci*	4	Zn-finger family TF; Hedgehog (Hh) signaling pathway; pattern formation and growth control; activator form needs *hh*	down	down	down
Trisomy female	*Jra*	2R	homologue of the mammalian transcription factor *Jun*; pleiotropic roles in the CNS; regulated by *bsk*	down	normal	down

Transcription factors are obtained from enrichment analysis of TF-binding motifs. This table lists the comparison groups, the TFs, the chromosomes that TFs located, the functions of candidate TFs, and the expression changes of the TFs in three kinds of aneuploidy.

## Data Availability

The sequencing data of *Inr-a* duplication *Drosophila* has been deposited in the Gene Expression Omnibus (GEO) database (https://www.ncbi.nlm.nih.gov/geo/, accession no. GSE180089, accessed on 11 August 2021) and the Sequence Read Archive (SRA) (https://www.ncbi.nlm.nih.gov/sra, accession no. SRP328302, accessed on 11 August 2021). The public data used in this study were downloaded from GSE46354, GSE41679, GSE53010 and GSE36736.

## Supplementary Materials

The following are available online at https://www.mdpi.com/article/10.3390/genes12101606/s1, Figure S1: GO and KEGG enrichment analysis of differentially expressed genes in three kinds of aneuploidy. Figure S2: Protein-protein interaction (PPI) networks of the common differentially expressed genes. Figure S3: Enrichment analysis of transcription factors for down-regulated genes in different aneuploidy groups. Figure S4: Gene expression of candidate transcription factors. Figure S5: Gene set enrichment analysis (GSEA) of signal transduction components and chromatin proteins. Table S1: Primers used in RT-PCR. Table S2: Validation of RNA-sequencing using relative quantitative PCR.
