# Supplementary material for: Modulation of Global Gene Expression by Aneuploidy and CNV of Dosage Sensitive Regulatory Genes
Source: Genes (Basel). 2021 Oct 12;12(10):1606. doi: 10.3390/genes12101606 (PMC8535535; doi:10.3390/genes12101606)
Supplement: Supplementary file 1 [file genes-12-01606-s001.zip › genes-1357002-supplementary.pdf]

Supplementary Material

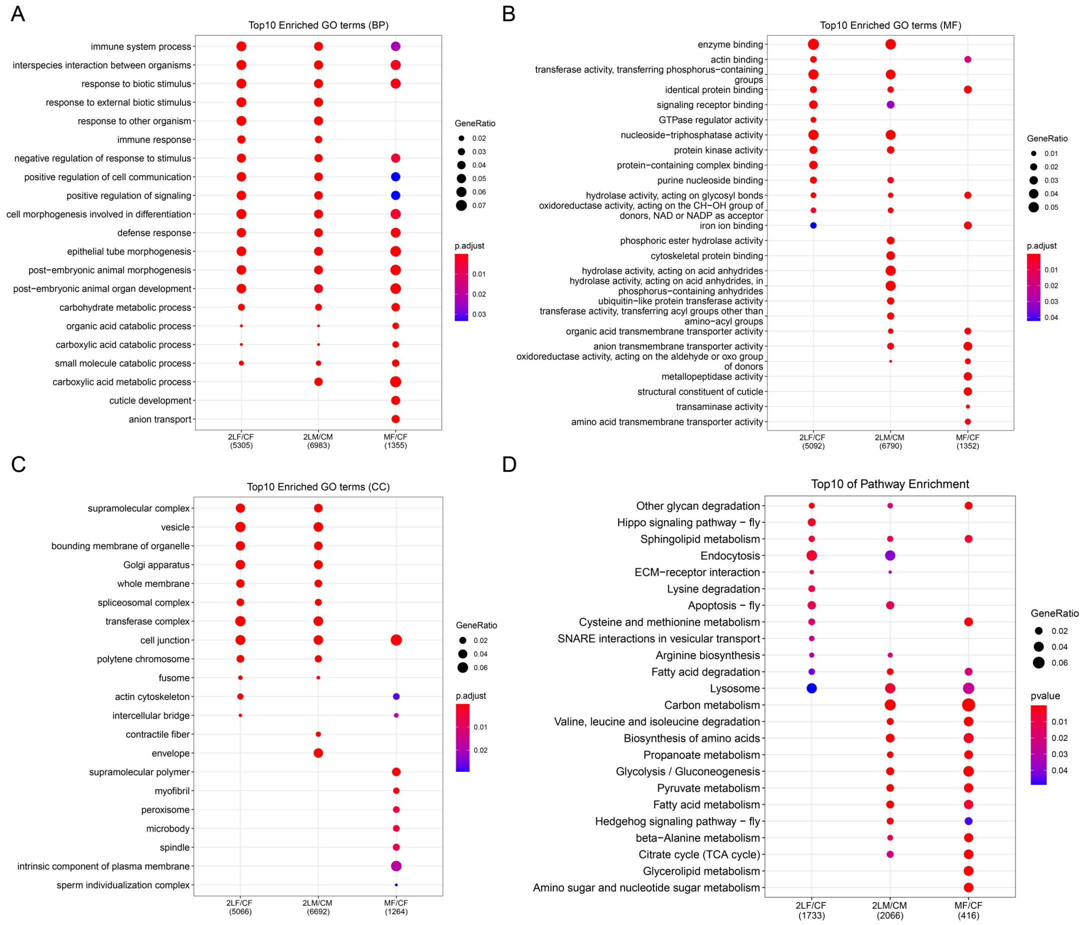

Figure S1. GO and KEGG enrichment analysis of differentially expressed genes in three kinds of aneuploidy. (A-C) Top 10 enriched GO terms in biological process (A), molecular function (B) and cellular component (C) of each aneuploidy. The size of the bubble represents the gene ratio, and the color represents adjusted p value as shown in the legend. (D) Top 10 enriched KEGG pathways of each aneuploidy. The size of the bubble represents the gene ratio, and the color represents p value as shown in the legend. The same enriched functions or pathways between different comparison groups are also shown. CF, wildtype female control; CM, wildtype male control; 2LF, trisomy 2L female; 2LM, trisomy 2L male; MF, metafemale.

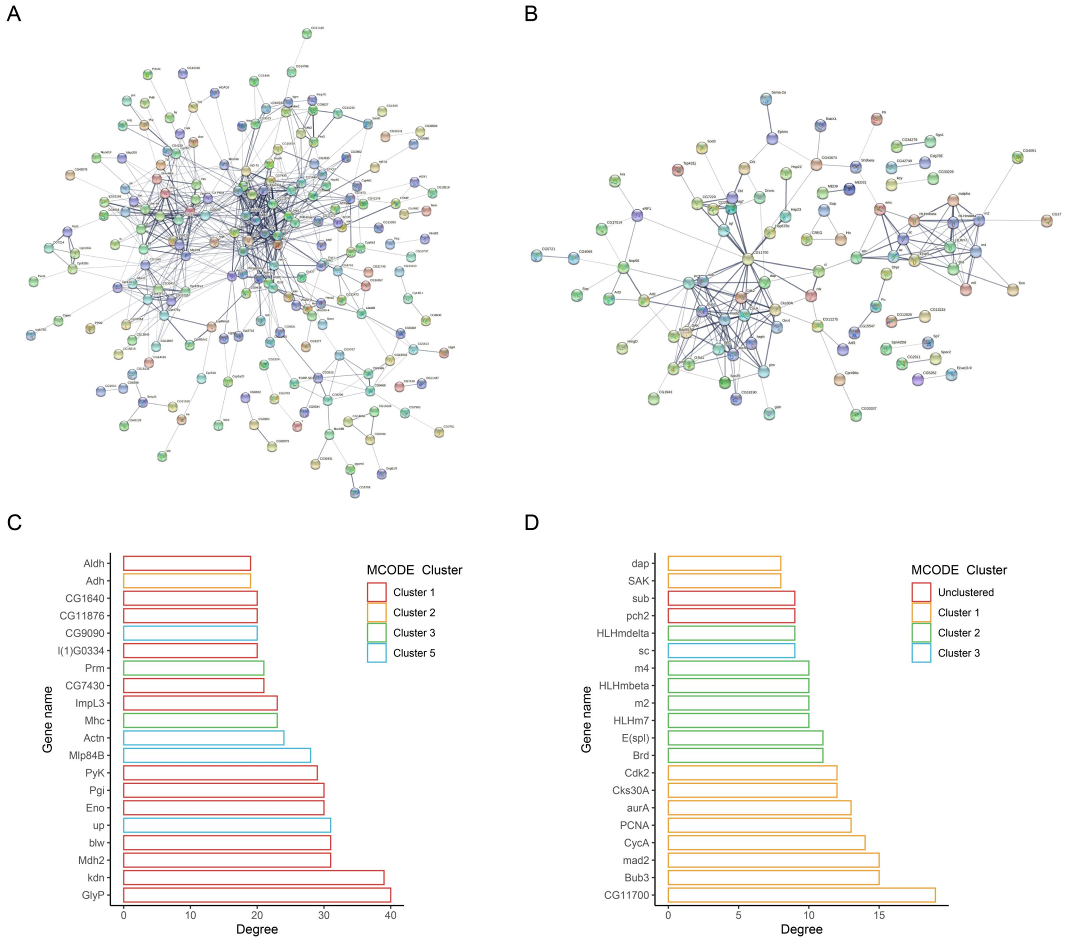

Figure S2. Protein-protein interaction (PPI) networks of the common differentially expressed genes. (A and B) PPI networks of common up-regulated (A) or down-regulated (B) DEGs. (C and D) Bar plots show the top 20 genes with the highest degrees in up-regulated (C) or down-regulated (D) PPI networks. The color of the bar represents the MCODE-module to which that gene belongs.

A

	logo	motif	NES	AUC	TF_highConf	nEnrGenes
1	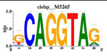	cisbp__M5265	5.18	0.07	zld	32
2	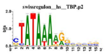	swissregulon__hs__TBP.p2	4.37	0.06	Tbp	46
3	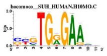	hocomoco__SUH_HUMAN.H10MO.C	4.23	0.06	Su(H)	8
4	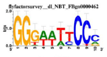	flyfactorsurvey__dl_NBT_FBgn0000462	4.06	0.06	dl	16
5	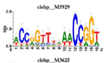	cisbp__M5929	4.04	0.06	gem	54
6	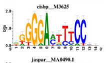	cisbp__M3625	4.00	0.06	Rel	15
7	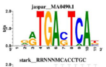	jaspar__MA0490.1	3.94	0.06	Jra	62
8	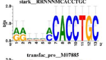	stark__RRNNNMCACCTGC	3.90	0.06	ac	14
9	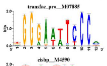	transfac_pro__M07885	3.84	0.06	Dif	16
10	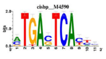	cisbp__M4590	3.76	0.06	kay	34

B

	logo	motif	NES	AUC	TF_highConf	nEnrGenes
1	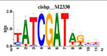	cisbp__M2330	6.28	0.04	pnr	746
2	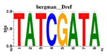	bergman__Dref	5.70	0.04	Dref	757
3	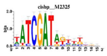	cisbp__M2325	5.31	0.03	BEAF-32	735
4	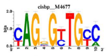	cisbp__M4677	5.08	0.03	kni	582
5	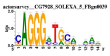	flyfactorsurvey__CG7928_SOLEXA_5_FBgn0039740	4.38	0.03	ZIPIC	219
6	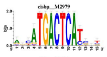	cisbp__M2979	3.87	0.03	cnc	106
7	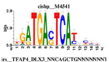	cisbp__M4541	3.79	0.03	Jra	112
8	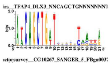	taipale_tf_pairs__TFAP4_DLX3_NNCAGCTGNNNNNNNNTAATTN_HT	3.49	0.03	crp	612
9	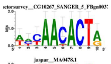	flyfactorsurvey__CG10267_SANGER_5_FBgn0037446	3.44	0.03	Zif	450
10	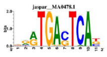	jaspar__MA0478.1	3.41	0.03	kay	103

C

	logo	motif	NES	AUC	TF_highConf	nEnrGenes
1	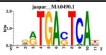	jaspar__MA0490.1	4.64	0.07	Jra	44
2	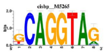	cisbp__M5265	4.45	0.07	zld	19
3	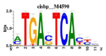	cisbp__M4590	4.36	0.07	kay	41
4	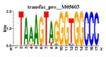	transfac_pro__M05603	4.23	0.07	Imd	37
5	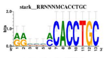	stark__RRNNNMCACCTGC	4.12	0.07	ac	10
6	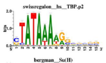	swissregulon__hs__TBP.p2	4.00	0.07	Tbp	51
7	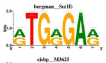	bergman__Su_H_	3.89	0.07	Su(H)	15
8	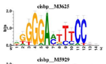	cisbp__M3625	3.87	0.07	Rel	17
9	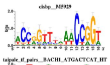	cisbp__M5929	3.78	0.07	gem	64
10	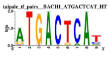	taipale_tf_pairs__BACH1_ATGACTCAT_HT	3.59	0.06	cnc	51

Figure S3. Enrichment analysis of transcription factors for down-regulated genes in different aneuploidy groups. The analysis is performed in all three aneuploidy groups (A), autosomal aneuploidy groups (B) and female aneuploidy groups (C) respectively. The over-represented motifs are ordered by normalized enrichment score (NES) with a threshold of 3, and the top 10 annotated transcription factors with high confidence are selected. The corresponding TF-binding motifs with the highest score are listed.

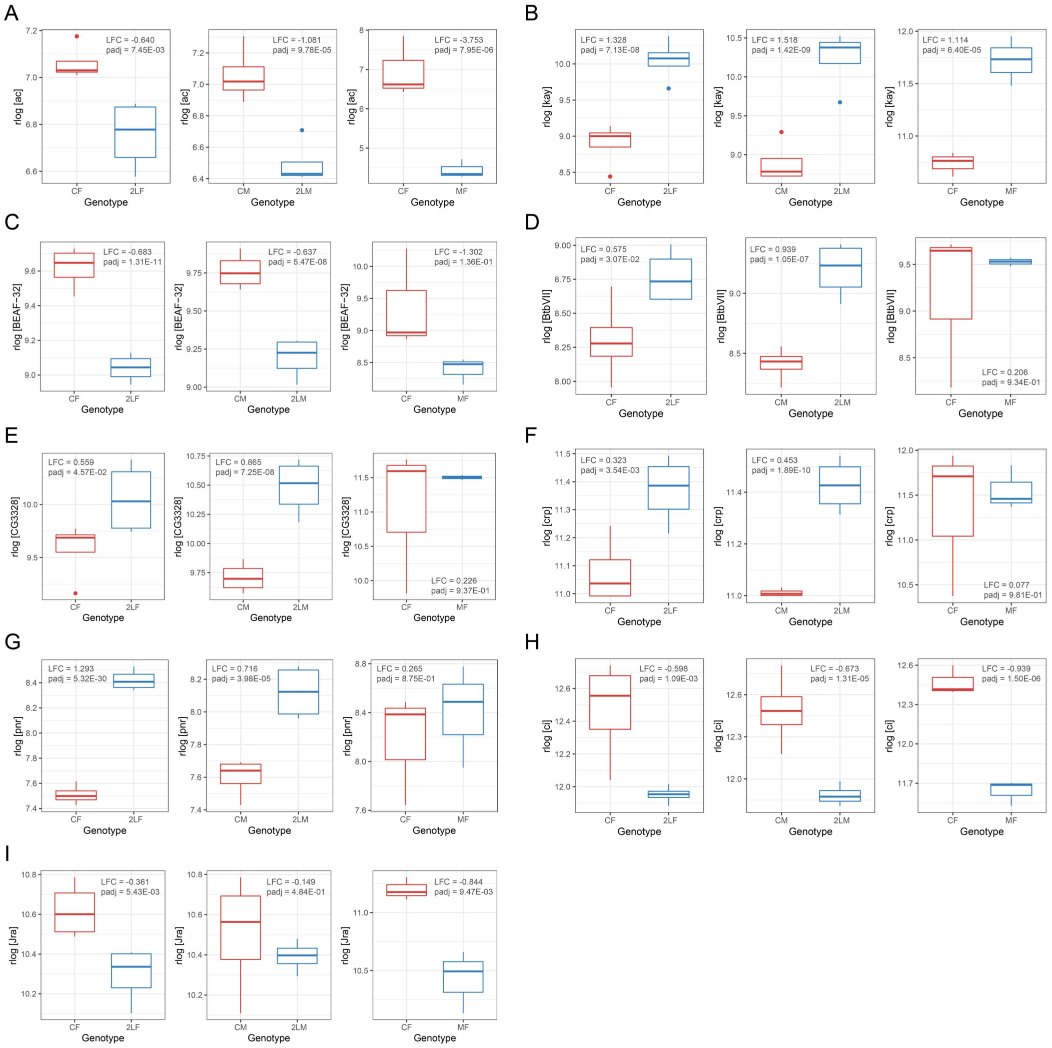

Figure S4. Gene expression of candidate transcription factors. (A-I) The boxplots show the expression levels of transcription factors *ac* (A), *kay* (B), *BEAF-32* (C), *BtbVII* (D), *CG3328* (E), *crp* (F), *pnr* (G), *ci* (H), and *Jra* (I). The gene expressions are shown using the regularized-logarithm transformation values.

Table S1. Primers used in RT-PCR

Genes	Chromosome	Left primer	Right primer
β-tubulin	2R	AGCTCAGCACCCTCTGTGTAAT	AGCTGGAGCGCATCAATGTGTA
CG43773	2L	GAGGAGTCCTGTACTATGCAAATC	CAGCGACTGGTACAAATGTAGT
jet	2L	GAACTCCACCAGCTTGCTTT	AACGAATGCGTCAACATCAC
magu	2R	TACATCTGCGTCCAAAGGC	GAATCCGAGAGAGATGGCTG
CG42694	2R	GGCTCCGATATCAAACCAATCT	GCTTACTCCAAGTTGCACAAAC
JIL-1	3L	ACCAGAAACGACCTTCCGAC	GTTGCATTAGTACGCGCAGG
oxt	3L	CCAACTTGTCGAGGGTCTTC	GGAAGCGATTCTCCACCATA
CG6293	3R	GCTATCCACCTTGCAGTATGT	CGAGAATTGTAGTGCCCAGTAG
Hsc70-4	3R	GCTTGATTGGTCGCAAGTTC	CGGTCTCCTTCATCTTGGTAAG
CG9577	X	CGTCGCCGTAAAGACAACCA	CACAGCCTGGGCGAAGTC
Karl	X	GAGAAGCTCGTGGGCAACAT	GTGTCGTAGTCCGTGTCCAGAA
Myb	X	TTGAAATGCGGTCCGATAAT	GGGACAGAACAAAAGCGGTA
sw	X	CAGGTTGGGATTGAACTTGG	TCGTACCACAACAACGAGGA
Ag5r2	X	GATTCTGGCCGGAGTACTTG	ACGATTCCAATCACAATGCC

Table S2. Validation of RNA-sequencing using relative quantitative PCR

Genes	Chromosome	RNA-seq	RT-PCR
CG43773	2L	0.759	0.944
jet	2L	0.892	0.783
magu	2R	0.742	0.781
CG42694	2R	0.837	1.148
JIL-1	3L	0.991	0.802
oxt	3L	0.885	1.063
CG6293	3R	1.174	1.420
Hsc70-4	3R	0.976	0.780
CG9577	X	0.810	1.044
Karl	X	0.985	1.048
Myb	X	1.795	1.711
sw	X	0.787	0.849
Ag5r2	X	1.236	1.781

The values in the table represent the relative expression levels of randomly selected genes in *Inr-a* duplication male compared with wild-type male *Drosophila*. For RT-PCR, three replicates per group were averaged. Overall, 70% of these genes were up- or down-regulated in the same direction.

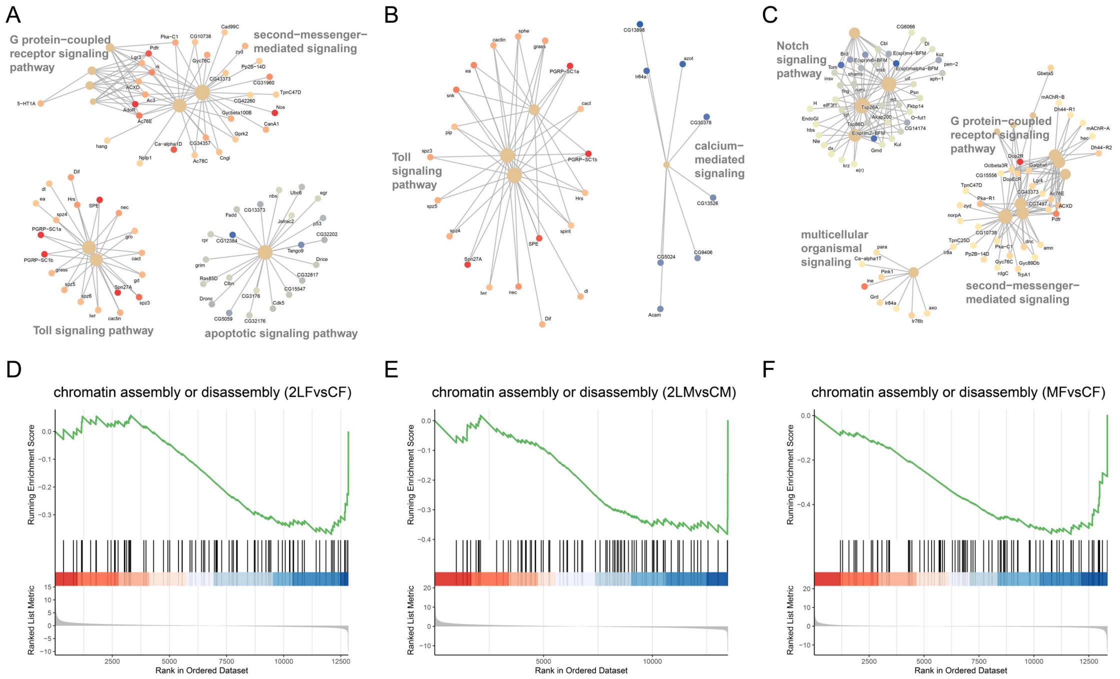

Figure S5. Gene set enrichment analysis (GSEA) of signal transduction components and chromatin proteins. (A-C) Gene-concept networks of signal transduction components in trisomy 2L female (A), trisomy 2L male (B) and metafemale (C) compared with normal diploid of the corresponding sex. The terms of p value < 0.05 are selected, and the similar term labels are simplified. Blue nodes represent down-regulated genes and red nodes represent up-regulated genes. (D-F) GSEA plots of chromatin proteins in trisomy 2L female (D), trisomy 2L male (E) and metafemale (F) compared with normal diploid of the corresponding sex. GSEA is used to evaluate the distribution trend of a predefined gene set in an ordered gene list. All genes are ordered by log2FoldChange (the bottom panel of each plot), and the distribution of genes included in the predefined gene set is shown in the middle panel. The green line in the top panel indicates the running sum statistic, and the maximum deviation from zero is enrichment sore. Only one term passed the filter with p value < 0.05. In three kinds of aneuploidy, the genes involved in chromatin assembly or disassembly tend to be distributed at the bottom of the total list.
